# Prognostic Value and Clinical Impact of ^18^FDG-PET in the Management of Children with Burkitt Lymphoma after Induction Chemotherapy

**DOI:** 10.3389/fmed.2014.00054

**Published:** 2014-12-16

**Authors:** Clément Bailly, Thomas Eugène, Marie-Laure Couec, Marion Strullu, Eric Frampas, Loïc Campion, Françoise Kraeber-Bodéré, Caroline Bodet-Milin

**Affiliations:** ^1^Department of Nuclear Medicine, University Hospital, Nantes, France; ^2^Department of Pediatric Oncology, University Hospital, Nantes, France; ^3^Department of Radiology, University Hospital, Nantes, France; ^4^U892, CNRS UMR 6299, CRCNA, INSERM, Nantes, France; ^5^Department of Biometrics, Cancer Center ICO René Gauducheau, Nantes, France

**Keywords:** Burkitt lymphoma, FDG-PET, pediatric lymphoma, induction chemotherapy, Deauville criteria

## Abstract

**Objective:** Burkitt lymphoma (BL) is a rare and aggressive form of B-cell lymphoma that is curable using intensive chemotherapy. Obtaining a complete response (CR) at the end of induction chemotherapy is a major prognostic factor. This study retrospectively evaluates the potential impact of ^18^FDG-PET in the management of children with BL after induction chemotherapy, and the prognostic performance of the Deauville criteria.

**Methods:** Nineteen children with BL treated according to the French LMB2001 protocol between 2005 and 2012 were included. ^18^FDG-PET and conventional imaging (CI) were performed after induction chemotherapy to confirm CR. ^18^FDG-PET was interpreted according to Deauville criteria with follow-up and/or histology as the gold standard.

**Results:** ^18^FDG-PET was negative in 15 cases, in agreement with CI in 9/15 cases. The six discordant cases confirmed to be negative by histology, were considered as true negative for ^18^FDG-PET. Negative predictive value (NPV) of CI and ^18^FDG-PET were 73 and 93%, respectively. The 5-year progression-free survival (PFS) was significantly higher in patients with negative ^18^FDG-PET than those with positive ^18^FDG-PET (*p* = 0.011).

**Conclusion:** ^18^FDG-PET interpreted using Deauville criteria can help confirm CR at the end of induction chemotherapy, with a prognostic impact on 5-year PFS. Its high NPV could limit the use of residual mass biopsy. Given the small size of our population, these results need to be confirmed by future prospective studies on a larger population.

## Introduction

Burkitt lymphoma (BL) is a highly aggressive B-cell non-Hodgkin lymphoma (NHL). It presents in three distinct clinical forms: endemic (areas of endemic malaria and early acquisition of Epstein–Barr virus infection), sporadic, and immunodeficiency-associated (1). In Europe and North America, BL is mostly sporadic and remains rare with an annual incidence of two per million children under 18 years. Nevertheless, it is the most frequent childhood NHL (30–40%) (2). The most common site of presentation is the abdomen (60–80%), with rapidly growing tumor masses, typically in the ileocecal region (3). Therefore, presenting symptoms include acute abdominal pain, distension, nausea and vomiting, and gastrointestinal bleeding. Authors consider bone marrow involvement occurs in roughly 20% of patients (4).

With the use of intensive multiagent chemotherapy, prognosis rates of children with BL have significantly improved over the past 25 years. Recent studies have shown that chemotherapy alone is effective in low, intermediate, and advanced stage disease (5–8) with 5-year event free survival ranging from 84% in advanced stage, to 92.5% in low-stage disease. Early response to chemotherapy was identified as an important prognostic factor by major United States and European childhood cancer groups, leading to adapted treatment protocols (7–9).

Positron emission tomography using 18F-fluoro-deoxy-glucose (^18^FDG-PET) is a functional imaging modality widely recommended for staging of FDG-avid lymphomas (10, 11). According to Lugano recommendations (10, 11), ^18^FDG-PET should also be used for response assessment in all FDG-avid histologies using the five-point scale (Deauville criteria). Nevertheless, while the prognostic value of the ^18^FDG-PET during treatment course has been strongly demonstrated in adult Hodgkin and aggressive lymphomas (12–15), only a few studies were conducted on ^18^FDG-PET in children lymphomas especially in BL (16–26). These studies showed that both nodal and extranodal manifestations of BL were detectable with this molecular imaging modality and suggested that ^18^FDG uptake was reversible after successful treatment (24–26).

This latter issue might be of great interest considering the importance of early response to chemotherapy in the prognosis and management of children BL (7–9). The need to achieve complete response (CR) after induction chemotherapy prior to deciding on further therapies raises the problem of residual mass depicted by conventional imaging (CI) after induction chemotherapy (27). A biopsy of these residual masses is recommended by most of treatment protocols, but it is invasive and only has value if positive. Given its potential to differentiate between necrotic or fibrotic tissue and viable tumor, ^18^FDG-PET could be an interesting method to characterize residual masses in BL and to avoid biopsy if negative.

The aim of this retrospective study was to demonstrate the potential impact of ^18^FDG-PET in the management of children with BL after induction chemotherapy. We also evaluated the prognostic performance of the^18^FDG-PET using the Deauville criteria in this pediatric type of lymphoma.

## Materials and Methods

### Patients

Nineteen children, diagnosed and treated for histologically proven sporadic BL, at University Hospital of Nantes between 2005 and 2012 were included. All of them were treated according to the French LMB 2001 protocol. Eighteen children were evaluated in first-line treatment. One patient was evaluated in first-line and during two relapses.

Patients with resected stage I and abdominal stage II disease received two courses of cyclophosphamide, vincristine, prednisone, and doxorubicin (COPAD) chemotherapy. Patients with central nervous system and/or bone marrow involvement received 7-day, low-dose, prophase cyclophosphamide, vincristine, and prednisone (COP) therapy. Induction therapy consisted of two cycles of fractionated COPAD and high-dose methotrexate (HD-MTX; COPADM). Consolidation included high-dose and continuous cytarabine with etoposide (CYVE). The other children received 7-day low-dose prophase COP. Their treatment then included two cycles of COPADM, two consolidation cycles of cytarabine and HD-MTX (CYM), and concluded with one maintenance phase of COPADM.

Written and informed consent was obtained from each patient and parents. The local ethics committee approved this study.

Population characteristics are summarized in Table 1.

**Table 1:** **Population and induction treatment characteristics**.

Patient no	Gender	Age at diagnosis	Stage	Induction treatment received before ^18^FDG-PET
1	M	6 years	III	COP – COPADM – COPADM – CYM
2	M	7 years	IV	COP – COPADM – COPADM – CYVE – CYVE
3	M	6 years	II	COPAD – COPAD
4	M	12 years	IV	COP – COPADM – COPADM – CYVE – CYVE
5	M	11 years	III	COP – COPADM – COPADM – CYM
6	M	14 years	IV	COP – COPADM – COPADM – CYVE – CYVE
7	M	8 years	III	COP – COPADM – COPADM – CYM
8	M	12 years	IV	COP – COPADM – COPADM – CYVE – CYVE
9	M	11 years	III	COP – COPADM – COPADM – CYM
10	F	9 years	III	COP – COPADM – COPADM – CYM
11	F	5 years	IV	COP – COPADM – COPADM – CYVE –CYVE
12	F	17 years	II	COP – COPADM – COPADM – CYM
13	M	4 years	III	COP – COPADM – COPADM – CYM
14	M	7 years	III	COP – COPADM – COPADM – CYM
15	M	3 years	IV	COP – COPADM – COPADM – CYM
16	M	13 years	III	COP – COPADM – COPADM – CYM
17	F	14 years	IV	COP – COPADM – COPADM – CYM
18	M	5 years	III	COP – COPADM – COPADM – CYM
				COP – CYVE –CYVE – RDA EPOCH – BEAM – Autograft
				COP – RDHAP – RIVA – RIVA – RIVA – Allograft
19	F	2 years	III	COP – COPADM – COPADM – CYM

*COP, cyclophosphamide, vincristine, and prednisone; COPAD, cyclophosphamide, vincristine, prednisone, and doxorubicin; COPADM, COPAD and methotrexate; CYM, cytarabine and methotrexate; CYVE, cytarabine and etoposide; RDA EPOCH, rituximab, dexamethasone, adryamicin, etoposide, prednisone, vincristine, cyclophosphamide, and doxorubicin; BEAM, carmustine, etoposide, cytarabine, and melphalan; RDHAP, rituximab, cisplatin, cytarabine, and dexamethasone; RIVA, rituximab, ifosfamide, vincristine, and doxorubicin*.

### Conventional imaging

Response assessment to induction chemotherapy was performed using CI as recommended in the LMB 2001 protocol. CI consisted, in addition to clinical examination, of chest X-ray, of contrast enhanced computed tomography (CT) (Sensation 16, Siemens; Light Speed VCT, GE Medical systems) and of ultrasound (US). MRI was performed for head and neck localizations or when meningeal involvement was suspected. All CI images were evaluated based on 1999 international workshop criteria (IWC) (28). Reviewing was performed in consensus by two experienced pediatric radiologists blinded to the results of other imaging studies but with knowledge of available clinical data. According to LMB 2001 protocol recommendations, only patients with CR according to 1999 IWC criteria were judged to be CI-negative after induction chemotherapy.

### ^18^FDG-PET procedure and analysis

In addition to the standard procedures, all children were examined with whole-body ^18^FDG-PET to evaluate response to induction chemotherapy. ^18^FDG-PET was performed after two courses of chemotherapy for stage II BL patients and after four or five courses for stage III–IV BL patients. In one patient evaluated at initial staging and during two relapses, ^18^FDG-PET was performed after two courses of chemotherapy in first-line and after four courses at relapses. ^18^FDG-PET results were not decisional in the patient management. ^18^FDG-PET could not be systematically performed at diagnosis because of the aggressiveness of the disease. Treatment had to be initiated rapidly and could not be delayed for imaging purposes.

Whole-body ^18^FDG-PET was acquired on a Discovery LS PET/CT imaging system (GE Medical Systems) 60–80 min after intravenous injection of 5–7 MBq/kg of ^18^FDG or on a mCT Biograph imaging system (Siemens) after intravenous injection of 3 MBq/kg of ^18^FDG. Children fasted at least 4 h before ^18^FDG injection and blood glucose was controlled prior to the injection. Images were reconstructed by OSEM iterative reconstruction algorithm (ordered-subset expectation maximization) with and without attenuation correction. All ^18^FDG-PET images were retrospectively reviewed on a dedicated workstation (Positoscope; Keosys, France).

^18^FDG-PET was interpreted visually by at least two nuclear medicine physicians with expertise in lymphoma imaging using the five-point scale (Deauville criteria), as recently recommended by Lugano’s Recommendations in Lymphoma (11). ^18^FDG-PET was interpreted as follows: 1 = no uptake above background, 2 = uptake equal to or lower than mediastinum, 3 = uptake between mediastinum and liver uptake, 4 = uptake moderately increased compared to the liver, and 5 = uptake markedly increased compared to the liver. Scales 1–3 were considered as ^18^FDG-PET negative and 4–5 as positive.

### Verification of findings

Surgical biopsy or resection was systematically performed in cases of residual mass on CI. To create a local standard of reference (SOR), all staging examinations, histopathology of biopsies and surgical specimens, and clinical data including the serial follow-up examinations were used for verification of the lesion status. Finally, the results of CI and ^18^FDG-PET were verified by an interdisciplinary tumor board.

### Statistics

Conventional imaging and ^18^FDG-PET results were compared to the status of the disease determined by SOR and classified as true positive or negative, and false positive or negative allowing determination of sensitivity (Se), specificity (Sp), positive predictive value (PPV), or negative predictive value (NPV).

The end point used to evaluate prognosis impact of ^18^FDG-PET was progression-free survival (PFS), defined as the time of diagnosis to disease progression, relapse, or death whatever the cause. Survival curves were calculated using Kaplan and Meier analysis. Differences between groups were analyzed using the Breslow test.

## Results

Nineteen children (5 female, 14 male) with histologically proven BL were included in this study. The median age was 9 years, and ranged from 2 to 17 years. All children were HIV-negative. A total of 21 ^18^FDG-PET were performed in addition to CI to confirm remission after the induction of chemotherapy. Population follow-up and imaging results are summarized in Table 2. Ten children (52%) did not reach CR on CI after induction chemotherapy. All residual masses were either resected or biopsied before therapeutic modification.

**Table 2:** **Population follow-up and imaging results**.

Patient no	Response to induction treatment by ^18^FDG-PET		Response to induction treatment by CI		Follow-up	
	Results	TO	Results	TO	Outcome	Time (months)
1	CR	TN	CR	TN	Alive	100
2	CR	TN	PR	FP	Alive	102
3	CR	TN	CR	TN	Alive	24
4	CR	TN	PR	FP	Alive	80
5	PR	TP	CR	FN	Deceased	9
6	PR	FP	PR	FP	Alive	86
7	CR	TN	CR	TN	Alive	42
8	CR	TN	CR	TN	Alive	40
9	PR	FP	PR	FP	Alive	56
10	CR	TN	CR	TN	Alive	56
11	CR	TN	PR	FP	Alive	46
12	CR	TN	CR	TN	Alive	57
13	CR	TN	PR	FP	Alive	34
14	PR	FP	PR	FP	Alive	31
15	CR	TN	CR	TN	Alive	34
16	CR	TN	PR	FP	Alive	29
17	PR	TP	PR	TP	Deceased	4
18	CR	FN	CR	FN	Relapse	
	PR	TP	CR	FN	Relapse	
	CR	TN	CR	TN	Alive	35
19	CR	TN	PR	FP	Alive	8

*CI, conventional imaging; ^18^FDG-PET, ^18^FDG-PET interpreted using Deauville criteria; TO, true outcome; TP, true-positive; TN, true-negative; FP, false-positive; FN, false-negative; CR, complete response; PR, partial response*.

### CI and ^18^FDG-PET interpretation

Conventional imaging was negative (CR without residual mass on CT) in 11 cases. In the 10 other cases, CT was positive and interpreted as partial response.

According to the gold standard, CI was considered as true negative in eight cases, false negative in three cases, true positive in one case and false positive in nine cases. The Se, Sp, PPV, and NPV of CI were, respectively, of 25, 47, 10, and 73%.

**Figure 1:**
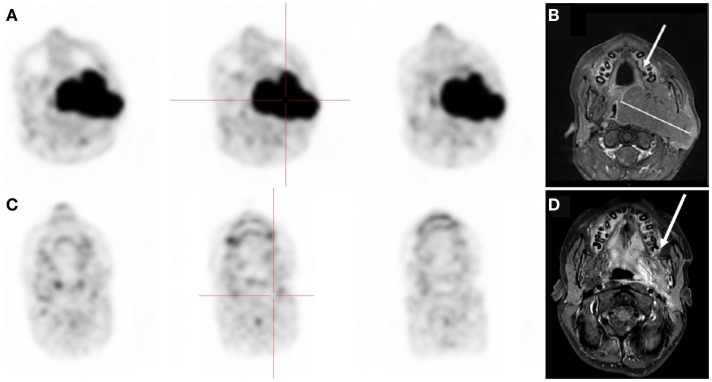
**Thirteen-year-old boy with stage III Burkitt’s lymphoma**. **(A,B)** ^18^FDG-PET and MRI at diagnosis showing oropharynx tumor. **(C,D)** Negative ^18^FDG-PET but residual disease on MRI after chemotherapy. Biopsy of the residual mass was negative.

According to the Deauville criteria, ^18^FDG-PET was negative in 15 cases and positive in 6 cases. ^18^FDG-PET was in agreement with CI in 9 of the 15 negative cases. The 6 discordant cases (patients no 2, 4, 11, 13, 16, and 19) presented residual masses without significant ^18^FDG uptake. Resection of these lesions revealed no viable tumor, with necrosis identified on histopathological examination (Figure 1).

One patient (patient no 18) was considered as a complete responder after induction treatment on both ^18^FDG-PET and CI. This patient experienced an early relapse three months after the end of treatment. For this analysis, we decided to consider ^18^FDG-PET and CI results as falsely negative.

**Figure 2:**
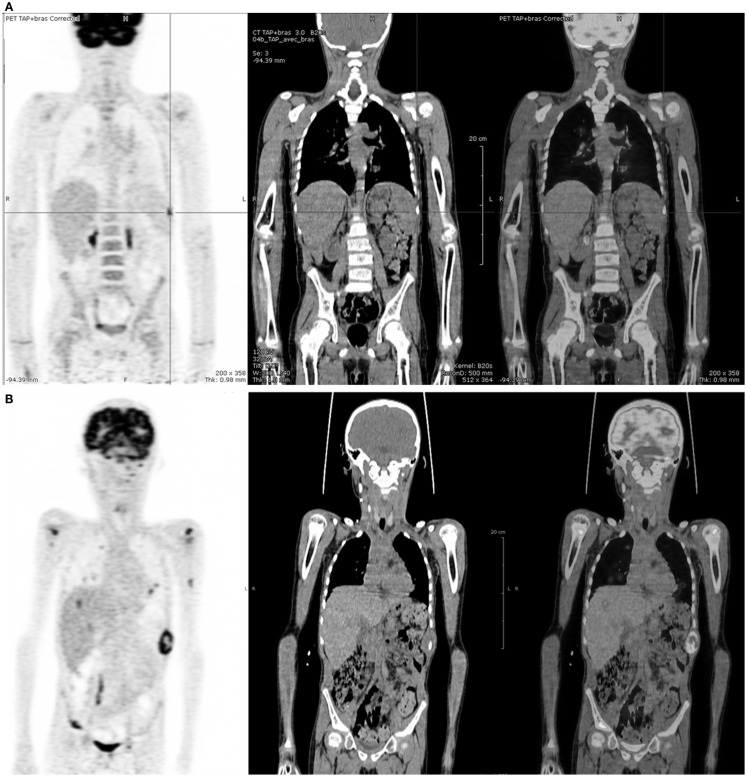
**Five-year-old boy with stage IV Burkitt’s lymphoma at relapse**. **(A)** ^18^FDG-PET after induction chemotherapy showing a nodular uptake on spleen whereas CI was negative. **(B)** ^18^FDG-PET 12 weeks later, showing disease progression.

^18^FDG-PET was positive in six cases, in agreement with CI in four cases. In the four concordant cases, only one (patient no 17) proved to be a true positive on histopathological analysis, showing residual tumoral cells. In the two discordant cases (patients no 5 and 18 at first relapse), an early progression was confirmed by follow-up and ^18^FDG-PET was considered as true positive (Figure 2).

According to the gold standard, ^18^FDG-PET was considered as true negative in 14 cases, false negative in 1 case, true positive in 3 cases, and false positive in 3 cases.

The Se, Sp, PPV, and NPV values of ^18^FDG-PET and CI were, respectively, 75, 82, 50, and 93 versus 25, 47, 10, and 73%. No significant difference was observed.

An overview of the diagnostic values of CI and ^18^FDG-PET is shown in Table 3.

**Table 3:** **Overview of diagnostic values obtained for ^18^FDG-PET and CI**.

	TP	FN	TN	FP	Sensitivity (%)	Specificity (%)	PPV	NPV
CI	1	3	8	9	25	47	10	73
^18^FDG-PET	3	1	14	3	75	82	50	93

*CI, conventional imaging; ^18^FDG-PET, ^18^FDG-PET interpreted using Deauville criteria; TP, true-positive; TN, true-negative; FP, false-positive; FN, false-negative; PPV, positive predictive value; NPV, negative predictive value*.

### Prediction of progression-free survival

Median follow-up of patients was 45 months (3–100 months). Of the 18 patients, one relapsed within 3 months and two died after a median delay of 4 months due to lymphoma progression. Except for the false-negative exam outlined above (patient no 18), neither progression nor relapse was observed in patients in the ^18^FDG-PET negative group.

**Figure 3:**
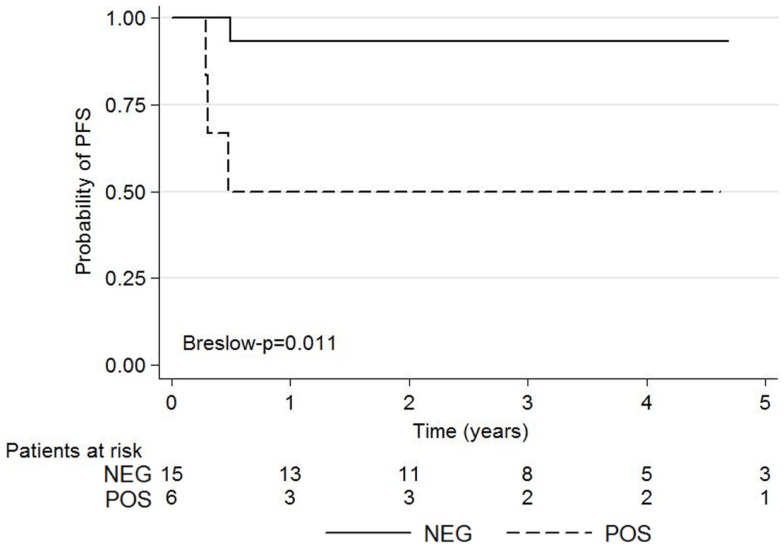
**Kaplan–Meier survival graphs show 5-year PFS according to negative and positive post-induction chemotherapy ^18^FDG-PET using the Deauville criteria**.

The Kaplan–Meier survival curves for 5-year PFS according to ^18^FDG-PET are shown in Figure 3.

The 5-year-PFS was significantly higher among patients with negative ^18^FDG-PET than those with positive ^18^FDG-PET (*p* = 0.011). Ninety-three percent (14/15) of patients with score 1, 2, or 3 on Deauville criteria did not experience relapse whereas 50% (3/6) of patients with score 4–5 relapsed or died.

**Figure 4:**
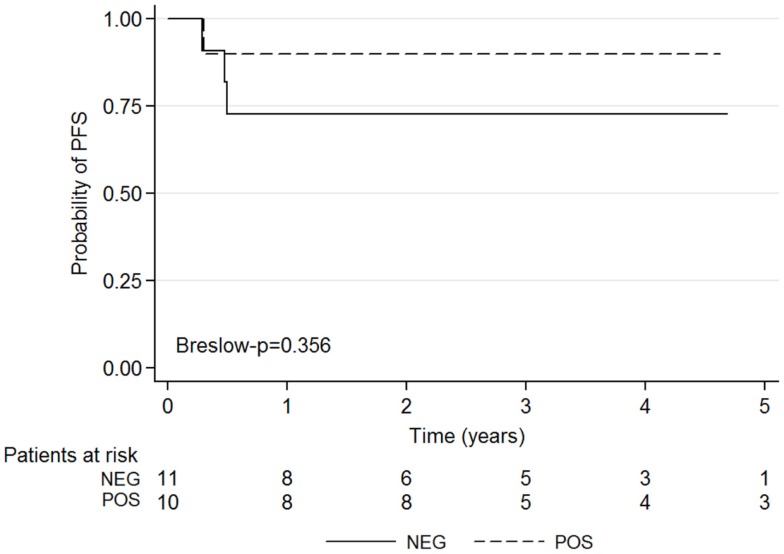
**Kaplan–Meier survival graphs show 5-year PFS according to negative and positive post-induction chemotherapy CI results**.

The Kaplan–Meyer survival curves showed no significant difference in PFS among patients with a positive or negative CI (*p* = 0.356) (Figure 4).

## Discussion

The few studies previously performed on pediatric lymphomas patients (16–22) demonstrated a good NPV of ^18^FDG-PET performed during treatment, with values ranging from 85 to 100% suggesting biopsy can be avoided if ^18^FDG-PET was negative. The recent pediatric study by Furth et al. (17) was conducted on 16 pediatric NHL patients and showed an overall NPV of 85.7%, rising to 100% when considering only BL (*n* = 7). Similarly, studies dedicated to pediatric BL by Karantanis et al. (23) and Riad et al. (25) reported a 100% NPV of ^18^FDG-PET after chemotherapy despite residual masses detected on CI. More recently, Carrillo-Cruz et al. analyzed the role of ^18^FDG-PET at the end of treatment using Deauville criteria. In this heterogeneous study including 13 children and 19 adults and different treatment schemes, the NPV of ^18^FDG-PET reached 100% (27). Our results are consistent with these data and strengthen the literature on a homogeneous population of pediatric BL, in which all residual lesions were biopsied or resected. Indeed, the NPV of ^18^FDG-PET was higher than 90 versus 75% for CI. NPV would have reached 100% if we did not decide to classify patient no 18 as FN (relapse 3 month after chemotherapy) despite the fact that he reached CR on both ^18^FDG-PET and CI, meaning no residual mass was detected. Considering our patients with residual masses detected by CI at the end of induction therapy without significant ^18^FDG uptake, none have experienced relapse during follow-up. These results suggest that in children BL, biopsy, or surgical resection of residual lesions depicted by CI after induction chemotherapy can be avoided when ^18^FDG-PET is negative.

As expected, considering the nature of ^18^FDG, most of the available studies have shown a high false positive rate, leading to a weak PPV. In the Bakhshi et al. study (22), PPV was 41.2% with only 7 patients considered as true positive on 17 positive ^18^FDG-PET scans. Riad et al. (25) described false-positive ^18^FDG uptake in four of 28 pediatric patients with abdominal BL. In these studies, abnormal ^18^FDG uptake was variously defined: uptake greater than background activity in surrounding tissue (19, 23, 24), or the mediastinal blood pool activity (17) or IHP criteria (18) for reference. In our study, ^18^FDG-PET images were interpreted with the current consensual set of criteria recently recommended by the “Lugano recommendations” (10, 11). We only reported three false positive results related to benign inflammatory processes detected by histopathological examination. If the use of Deauville criteria slightly improves PPV compared to previously described criteria, the PPV remained poor. The PPV of CI is lower in our study than in previously published reports (18, 19, 22). This lower CI PPV can be easily explained: because surgical biopsy or resection was systematically performed in cases of residual masses on CI, only patients with CR according to 1999 IWC criteria (28) were judged to be CI-negative. In other studies, CR and/or unconfirmed complete responders (i.e., with residual masses on CI) were considered to be CI-negative. However, PPV remained poor according to each modality, and biopsy remained essential in ^18^FDG-PET and CI positive cases.

Recent studies in adult NHL (29) and HL (30) reported high-prognostic value of interim ^18^FDG-PET (after two or four courses of chemotherapy). These kinds of results were not described by any previous studies of pediatric NHL. Furth et al. (17) revealed no significant difference in PFS neither for interim CI, nor for ^18^FDG-PET, nor for semi-quantitative analysis using delta SUVmax in 18 children with lymphomas including 7 BL. In this study, ^18^FDG-PET was performed after two cycles of chemotherapy regardless of the therapeutic scheme or stage of disease, and ^18^FDG-PET were interpreted visually using IHP criteria. In the Bakhshi et al. study (22), response at interim ^18^FDG-PET or CI did not predict PFS or overall survival in 34 patients with non-lymphoblastic lymphomas including 28 B-cell lymphomas. In Carrillo-Cruz’s study (27), the Deauville criteria (score ≥4 as positive) did not allow to predict outcome accurately in a heterogeneous population of BL when quantitated at the end of treatment. On the contrary, in our study, the use of Deauville criteria (score ≥4 as positive) improved specificity and PPV and ^18^FDG-PET was predictive of outcome (*p* = 0.011) when performed in an earlier setting (after induction chemotherapy).

A very recent study on adult diffuse large B-cell lymphoma, showed that if visual analysis can be employed reliably, computation of semi-quantitative analysis (ΔSUVmax) leads to better outcome prediction and better reproducibility among observers (29) in interim ^18^FDG-PET (after two and/or four courses of chemotherapy). In the Carrillo-Cruz study (27), NPV reached 100% when ΔSUVmax was <66% of the initial value at the end of treatment but the prognosis value of the semi-quantitative analysis was not studied. Unfortunately, as our study was retrospective, we were unable to complete our data by semi-quantitative analysis for two reasons: unavailable baseline ^18^FDG-PET for some of our patients with very aggressive disease and media-storage degradation for patients included from 2005 to 2008.

## Conclusion

Our study confirms that ^18^FDG-PET’s very high NPV could limit the use of biopsy of residual masses in sporadic pediatric BL. Our results also suggest that ^18^FDG-PET interpreted using Deauville criteria can help confirm early CR at the end of induction chemotherapy with prognostic impact on 5-year PFS. However, considering the poor PPV, biopsy remains essential to characterize ^18^FDG-PET positive residual masses. Nevertheless, given the small size of our study population and the rarity of this lymphoma, future prospective studies on a larger population of children, probably as part of a multicenter study, are highly warranted.

## Conflict of Interest Statement

The authors declare that the research was conducted in the absence of any commercial or financial relationships that could be construed as a potential conflict of interest.
